# Supplementary material for: Scaling the effects of ocean acidification on coral growth and coral–coral competition on coral community recovery
Source: PeerJ. 2021 Jul 13;9:e11608. doi: 10.7717/peerj.11608 (PMC8284307; doi:10.7717/peerj.11608)
Supplement: Supplemental Information 1 [file peerj-09-11608-s001.docx]

*The following supplement accompanies the article:*

**Scaling the effects of ocean acidification on coral growth and coral-coral competition on coral community recovery**

Nicolas R. Evensen, Yves-Marie Bozec, Peter J. Edmunds, Peter J. Mumby

*Corresponding author: nicolas.r.evensen@gmail.com

**Supplementary Material**

Table S1. Demographic parameters for each coral genus based on empirical and experimental data collected in Mo'orea, and from values reported in peer-reviewed publications. Model timesteps are 6 months.

| **Parameter** | *Acropora* | *Montipora* | *Pocillopora* | *Porites* |
| --- | --- | --- | --- | --- |
| Mean recruitment following adjustments (individuals.m^-2^ .timestep^-1^)^†^ | 0.03-0.25 | 0.24-2.42 | 0.96-4.15 | 1.2-3.9 |
| Linear extension (cm.yr^-1^) under ambient conditions | 5^a,b^ | 3^c^ | 3.2^d^ | 1.3^b,e^ |
| Partial colony mortality (% of colonies per time step experiencing a reduction in colony size)^‡^ | 0.15 | 0.15 | 0.45 | 1.5 |
| Whole colony mortality (% of colonies per time step)^‡^ | 2.5 | 2.5 | 2.5 | 1.5 |

^†^Based on field data of recruitment to tiles (Edmunds 2018), and adjusted so that model simulations matched field observations

^‡^Based on field data collected by Kayal et al. (2018) from LTER1

^a^Stimson, 1985; ^b^Morgan & Kench, 2012; ^c^Browne, 2012; ^d^Evensen & Edmunds, 2016; ^e^Lough & Barnes, 2000

Table S2. Sensitivity of individual coral genera to elevated pCO_2_, with values indicating the percent decrease in linear growth under OA relative to ambient conditions. Details of the experiments Study location and type from which the sensitivity of each genera to elevated pCO_2_ was obtained are also provided.

| **Parameter** | *Acropora*^a^ | *Montipora*^b^ | *Pocillopora*^c^ | *Porites*^d^ |
| --- | --- | --- | --- | --- |
| Sensitivity to elevated pCO_2_ under OA | 37 | 43 | 30 | 0 |
| Mean pCO_2_ levels in study (µatm) | 1795 | 972 | 1033 | 804 |
| Study location | Eilat, Gulf of Aqaba | Mo'orea, French Polynesia | Mo'orea, French Polynesia | Mo'orea, French Polynesia |
| Study type | Tank experiment | Tank experiment | Flume experiment | Tank experiment |

^a^Horwitz et al., 2017; ^b^Evensen, Edmunds & Sakai, 2015; ^c^Evensen & Edmunds, 2016; ^d^Edmunds, Brown & Moriarty, 2012

**
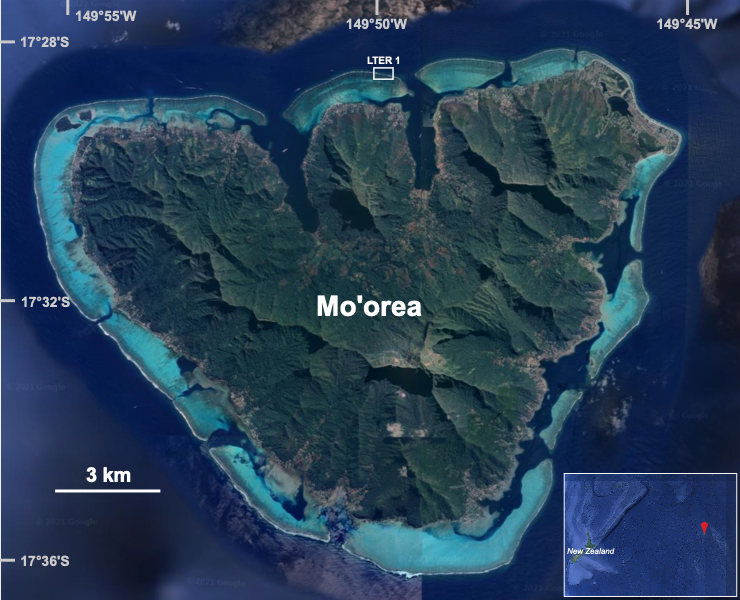
Figure S1.** Map of the study site in Mo'orea, French Polynesia, with the fore reef study site (LTER1) outlined by the white box. Inset, location of Mo'orea (red marker) within the South Pacific basin. Photo credits: Google Earth.

**Supplemental Methods**

The calculations in the code below used to estimate contact between colonies in model simulations were initially established to determine contact between colonies in photoquadrats from the outer reefs of Mo’orea. First, the radius and distance between the centres of each colony were measured in each quadrat or cell. In turn, this indicated which colonies were within 1.5 cm of one another in each quadrat/cell, and that would therefore grow into contact after an anticipated radial extension of 6 months (i.e., one model time step). For the colonies within 1.5 cm of one another, the distance between the centre of the colonies and the radii of the colonies after 6-months growth were used to calculate the area of a theoretical triangle, using Heron’s formula (Weisstein 2021), created by interacting pairs of colonies (Fig. S2a, shown below). Then, the height of the triangle was calculated, which represented half the length of the chord created by the intersection of the two colonies (Fig. S2b, shown below). The length of the chord then was used to calculate the length of the arc for each coral that was in contact with the other colony, which was then converted to a percentage of each colony’s perimeter in contact with the other coral. These measurements were replicated for corals that were within 1.5 cm of multiple colonies. We then calculated the average percent contact between all colonies within a quadrat/cell, with the sum of the area of all colonies providing a measure of total coral cover for each quadrat/cell. Together, these were used to assess the relationship between coral cover and percent contact between corals.


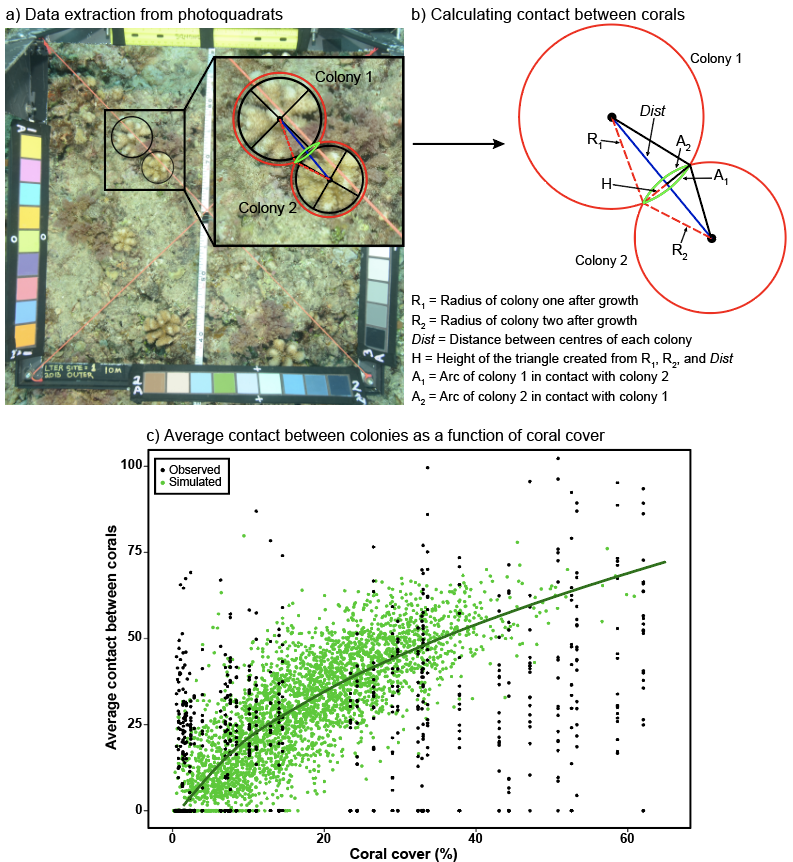


**Figure S2.** From Figure 1 in the main manuscript: (a) Measurements of the diameters and circumferences of two *Pocillopora* colonies (black lines and circles) and their anticipated growth over 6 months (i.e., one model time step) (red circles), based on growth rates measured in experiments in Mo'orea (Evensen & Edmunds, 2016). The blue line represents the distance between colony centres, with red dashed lines representing the anticipated radii of each colony after growth and the green lines representing the section (arc) of each colony in contact with the other colony after growth. (b) Using the distance between colony centres (*Dist*) and anticipated radii of each colony after growth (R_1_ and R_2_) to calculate the height of the triangle (H), which was then used to calculate the length of the contact arc between corals (A_1_ and A_2_). This approach was used to parameterize competition between coral colonies in the model using the following code in Matlab:

%_____________________________________________________________

%

% GENERATING RANDOM CORALS IN A CELL TO ASSESS CORAL CONTACT

% AS A FUNCTION OF CORAL COVER

%_____________________________________________________________

%

% Defining the function used to calculate percent contact between colonies as a function of their % size and distance from one another.

function [C1,C2]=calculate_contact(dist,radius1,radius2)

semiperimeter=(radius1+radius2+dist)/2; %needed for Heron's formula (area calculation % below) - using triangle created by radii of two intersecting % colonies and the distance between their centers

area=sqrt(semiperimeter.*(semiperimeter-dist).*(semiperimeter-radius1).*(semiperimeter-radius2)); %calculate area of the triangle using the distance between centers and radii of two ________% circles/colonies in question

height=(area*2./dist); % Calculate the height, which is half the length of the chord - need % chord to calculate length of contact arc between colonies

contact_arc=radius1.*asin(2*height./(radius1)); % calculate contact arc for 1st coral

contact_arc2=radius2.*asin(2*height./(radius2)); % calculate contact arc for 2nd coral

C1=(contact_arc./(2*pi*radius1))*100; % use circumference to calculate percent of the % circumference (arc) that is in contact

C2=(contact_arc2./(2*pi*radius2))*100; % same as above but for 2nd coral

% List of variables

growth=1.5; %linear growth of a coral during a 6-month timestep

dmax=35; % max. number of colonies in a cell

sizemax=291; % max observed colony size - based on photoquadrat data from LTER1

sizemin=0.22; %min size based on photoquadrats

nsimul=100; % no. of simulations for each coral density

xmax=50; %number of cells on x-axis

ymax=50; %number of cells on y-axis

m = [1.269 2.389 10.393 34.773 65.497]; %mean size of observed corals for each year

v = [0.933 3.976 100.088 1100.636 3208.357]; %variance in size of observed coral for each year

%Define matrices

CONTACT=zeros(dmax-1,nsimul); % average contact over all corals

COVER=zeros(dmax-1,nsimul); % total coral cover in simulated quadrat

for Density=2:dmax %Set number of colonies being simulated

for simul=1:nsimul %Set number of simulation for each colony density

Q=zeros(Density,3); %create matrix for each information for each quadrat

for coral=1:Density % for each coral

Q(coral,1) = round(rand(1)*xmax); %populate matrix with x-coordinates for each coral

Q(coral,2) = round(rand(1)*ymax); %populate matrix with y-coordinates for each coral

select=randi(5); %randomly select a year’s mean and variance

mu = log((m(select)^2)/sqrt(v(select)+m(select)^2)); %calculate log mean

sigma = sqrt(log(v(select)/(m(select)^2)+1)); %calculate log variance

s = lognrnd(mu,sigma); % produce random size (cm^2^) for each coral based on lognormal % distribution

s(s > sizemax) = sizemax; %Converts all values generated above max size into upper size % limit

s(s < sizemin) = sizemin; % Converts all values generated above min size into lower size % limit

Q(coral,3) = s; % Populate matrix with sizes

end

COVER(Density-1,simul)=100*(sum(Q(:,3),1)/(xmax*ymax)); %create sum of all corals in % each quadrat to have total coral cover in each quadrat

INDCONTACT=zeros(Density,Density-1); % matrix to store percent contact for each coral

for c1=1:Density %take the first coral in a quadrat

x1=Q(c1,1); y1=Q(c1,2); radius1=sqrt(Q(c1,3)/pi)+growth; %calculate location + radius % of the coral

neighbours=1:Density; %assess all other corals in the quadrat

neighbours(neighbours==c1)=[]; %remove the focal coral from that list of corals

for i=1:length(neighbours) % no. of neighbours

c2=neighbours(i); % take the first neighbour in the list

x2=Q(c2,1); y2=Q(c2,2); radius2=sqrt(Q(c2,3)/pi)+growth; % calculate its position and % size

dist=(sqrt((x2-x1)^2+(y2-y1)^2)); % calculate distance between the focal coral and 'c2'

if dist<=0

continue

elseif radius1 > dist+radius2 || radius2 > dist+radius1

INDCONTACT(c1,i) = 100;

else

if dist < radius1+radius2 % only calculate contact if the distance between them is % smaller than their combined radii (otherwise contact is 0%)

[INDCONTACT(c1,i),~]=calculate_contact(dist,radius1,radius2); % calculate the % contact between corals

end

end

end

end

% When contact has been calculated for all colonies.

CUMUL_INDCONTACT = sum(INDCONTACT,2); %For colonies in contact with multiple colonies, sum contact

CUMUL_INDCONTACT(CUMUL_INDCONTACT>100) = 100; %Ensures contact for a colony cannot exceed 100%

CONTACT(Density-1,simul) = mean(CUMUL_INDCONTACT); %Calculate mean contact for all colonies in a cell

end

end

ModelCompetition=fitlm(COVER.^0.5,CONTACT);

ModelCompetition =

*Linear regression model:*

*y ~ 1 + x1*

*Estimated Coefficients:*

*Estimate SE tStat pValue*

*(Intercept) -12.367 0.47836 -25.852 1.0975e-134*

*x1 10.645 0.1166 91.296 0*

*Number of observations: 3400, Error degrees of freedom: 3398*

*Root Mean Squared Error: 9.95*

*R-squared: 0.71, Adjusted R-Squared 0.71*

*F-statistic vs. constant model: 8.33e+03, p-value = 0*

**References**

Browne NK (2012) Spatial and temporal variations in coral growth on an inshore turbid reef subjected to multiple disturbances. Marine Environmental Research 77:71−83. doi: https://doi.org/10.1016/j.marenvres.2012.02.005

Edmunds PJ (2018) MCR LTER: Coral Reef: Long-term Population and Community Dynamics: Corals, ongoing since 2005.

Edmunds PJ, Brown D, Moriarty V (2012) Interactive effects of ocean acidification and temperature on two scleractinian corals from Moorea, French Polynesia. Global Change Biology 18:2173–2183. doi: 10.1111/j.1365-2486.2012.02695.x

Evensen NR, Edmunds PJ (2016) Interactive effects of ocean acidification and neighboring corals on the growth of *Pocillopora verrucosa*. Marine Biology 163:148. doi: 10.1007/s00227-016-2921-z

Evensen NR, Edmunds PJ, Sakai K (2015) Effects of pCO2 on spatial competition between the corals *Montipora aequituberculata* and *Porites lutea*. Marine Ecology Progress Series 541:123–134. doi: 10.3354/meps11512

Horwitz R, Hoogenboom MO, Fine M (2017) Spatial competition dynamics between reef corals under ocean acidification. Scientific Reports 7:40288. doi: 10.1038/srep40288

Kayal M, Lenihan HS, Brooks AJ, Holbrook SJ, Schmitt RJ, Kendall BE. 2018. Predicting coral community recovery using multi-species population dynamics models. *Ecology Letters* 21:1790–1799. DOI: 10.1111/ele.13153.

Lough JM, Barnes DJ (2000) Environmental controls on growth of the massive coral *Porites*. Journal of Experimental Marine Biology and Ecology 245:225–243. doi: https://doi.org/10.1016/S0022-0981(99)00168-9

Morgan KM, Kench PS (2012) Skeletal extension and calcification of reef-building corals in the central Indian Ocean. Marine Environmental Research 81: 78–82. doi: https://doi.org/10.1016/j.marenvres.2012.08.001

Stimson J (1985) The effect of shading by the table coral *Acropora hyacinthus* on understory corals. Ecology 66: 40–53. doi: https://doi.org/10.2307/1941305

Weisstein EW. 2021.“Heron’s Formula.” From MathWorld--A Wolfram Web Resource. https://mathworld.wolfram.com/HeronsFormula.html
